# Accumulation of Antioxidative Phenolics and Carotenoids Using Thermal Processing in Different Stages of *Momordica charantia* Fruit

**DOI:** 10.3390/molecules28031500

**Published:** 2023-02-03

**Authors:** Ji Hye Kim, You Jin Lim, Shucheng Duan, Tae Jung Park, Seok Hyun Eom

**Affiliations:** Department of Smart Farm Science, College of Life Sciences, Kyung Hee University, Yongin 17104, Republic of Korea

**Keywords:** antioxidant, bitter gourd, carotenoid, fruit stage, polyphenol, thermal processing

## Abstract

The bitter taste of *M. charantia* fruit limits its consumption, although the health benefits are well known. The thermal drying process is considered as an alternative method to reduce the bitterness. However, processing studies have rarely investigated physiochemical changes in fruit stages. The antioxidant activities and physiochemical properties of various fruit stages were investigated using different thermal treatments. The color of the thermally treated fruit varied depending on the temperature. When heat-treated for 3 days, the samples from the 30 °C and 90 °C treatments turned brown, while the color of the 60 °C sample did not change significantly. The antioxidant activities were increased in the thermally processed samples in a temperature-dependent manner, with an increase in phenolic compounds. In the 90 °C samples, the 2,2-diphenyl-1-picrylhydrazyl radical scavenging activity presented a 6.8-fold higher level than that of nonthermal treatment in mature yellow fruit (S3), whereas the activity showed about a 3.1-fold higher level in immature green (S1) and mature green (S2) fruits. Regardless of the stages, the carotenoid content tended to decrease with increasing temperature. In terms of antioxidant activities, these results suggested that mature yellow fruit is better for consumption using thermal processing.

## 1. Introduction

*Momordica charantia*, commonly known as bitter gourd, belongs to the Cucurbitaceae family and contains pharmaceuticals that are employed in traditional Asian medicines. The fruit of *M. charantia* is widely cultivated in tropical and subtropical climates, such as India, China, and Thailand for vegetables and medicinal usage [1]. The fruit are widely used with not only fresh salad and juice but also pre-boiling, drying, stir-frying, and frying to reduce their bitter flavor [2]. In addition, *M. charantia* has been used as a source of medicine to treat cough-, liver heating-, anthelmintic-, and diabetes-related diseases [3,4,5]. In particular, its fruit contains health-beneficial bioactive compounds such as charantin and it is particularly attractive for use in food and pharmaceuticals. In addition, it contains plenty of vitamin C, phenolic acids, and carotenoids, which have been considered antioxidants in food ingredients [6].

Despite these health advantages, its consumption has been limited due to a strong bitter taste, especially in raw fruit. Therefore, numerous attempts are being performed to reduce the taste while maintaining the health benefits. Heat treatments such as baking, roasting, and pressure cooking are known to reduce bitterness [7]. It is known that saponins, including momodicoside F, which contribute to the bitter taste in *M. charantia*, are also reduced by heat treatment [8]. Furthermore, hot-air drying is one of the most commonly employed heat treatments that alters not only the physical properties (such as hardness) but also the chemical properties (such as polyphenol decomposition) of food materials. Heat treatment increases taste preferences by promoting nonenzymatic reactions in food or inducing a change in flavor components [6,7,8,9,10,11,12,13]. Heat treatment can cause an increase in phenolic compounds and antioxidant activity in various fruit crops such as citrus, persimmon peel, and eggplant fruit [10,14,15]. On the other hands, phenolic compounds and antioxidant activity were decreased by heating in some crops such as olive, persimmon flesh, and plum [10,16,17]. According to Choi et al. [18], *M. charantia* fruit roasted at 200 °C for 15 min showed similar antioxidant activity compared to the unroasted sample, but the flavan-3-ol and phenolic acid contents were about 1.4 times higher. Furthermore, Ng and Kuppusamy [19] found that heat treatments such as microwave heating and boiling were effective in increasing the antioxidant activity of *M. charantia* extract. Several studies have focused on the biological activities and variations in bioactive compounds during different thermal processing methods; however, current scientific information does not explain how these changes in the fruit occur at different hot-air drying temperatures.

*M. charantia* changes morphological and physicochemical characteristics depending on the growth stage. The fruit turns yellow as it ripens and the bitterness of the fruit decreases and the sweetness increases at this stage [20,21]. However, the fruit shows signs of decay and splitting, including cracking or bursting, causing it to be impossible for consumption as fresh fruit. Therefore, *M. charantia* is usually used in its mature green skin stage. Phenolic compounds can be increased or decreased depending on the crops, maturity, and processing methods. It was reported that the phenolic content decreased as the olive fruit matured, while it increased as soybean seed matured [9,22]. Moreover, the total phenolic content decreased during thermal processing in the flesh of persimmon fruit with the decrease in antioxidant activity, while it increased in the peel [10]. In a previous study, it was discovered that the polyphenolic compounds and antioxidant capacities of *M. charantia* were altered at various maturity stages [23,24]. It is reported that several phenolic compounds increased as *M. charantia* fruit matures [20,24]. Moreover, certain studies have shown the comparison between mature and immature fruits or leaves in different cultivars [25,26]; however, thermal processing at the different stages has not been studied in terms of the processing and antioxidant effects.

Therefore, the present study aimed to investigate the changes in antioxidant activities, phenolic compounds, carotenoids, and chlorophylls according to thermal treatments in the growth stage of *M. charantia* fruit. Furthermore, we have analyzed the major antioxidants using Pearson correlation coefficiency analysis between antioxidant activities and the active compounds.

## 2. Results

### 2.1. Changes of Color during Thermal Process

**Figure 1:**
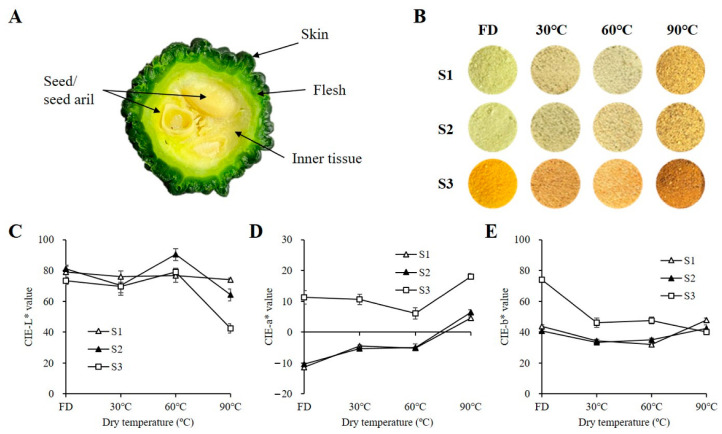
Morphological characteristics of *M. charantia* (**A**) cross section at the central part of *M. charantia*, (**B**) visual color, and (**C**) their CIE L*, (**D**) a*, and (**E**) b* values of *M. charantia* (ground powder) at different stages after thermal processing for 3 days. FD indicates freeze dry. S1, S2, and S3 indicate immature green fruit about 15 days after fertilization (DAF), mature green fruit about 25 DAF, and mature yellow fruit about 30 DAF.

Figure 1 shows the morphological characteristics of *M. charantia* with different maturity stages, presenting stage 1 (S1) as immature green fruit about 15 days after fertilization (DAF), stage 2 (S2) as mature green fruit at about 25 DAF, and stage 3 (S3) as mature yellow fruit at about 30 DAF. The treatments for each temperature at different stages were heat treated for 3 days and the freeze-dried samples were used as control. In the nonthermal process, the S1 and S2 fruits presented a light green color, while the fruits turned greenish yellow after 90 °C temperature treatment. S3 was yellow in nonthermal conditions and turned dark brown after 90 °C temperature treatment. The fruits dried at 60 °C in all stages showed a lighter color than those in the 30 °C and 90 °C temperature treatments. Figure 1C presents the CIE-Lab color values of these samples. The L* values of *M. charantia* in all stages were similar at temperatures below 60 °C. Furthermore, the L* value of S3 fruit decreased and was lower than that of S1 and S2 at 90 °C. The increasing a* value representing the color varied from greenness to redness. The increased a* values were observed as the drying temperature was enhanced regardless of the growth stages. The values of S3 ranged from 7.2 to 19.1 and were higher than those of S1 and S2 at all drying temperatures. The increasing b* value representing the color varied from blueness to yellowness. The b* values were maintained regardless of drying temperatures in S1 and S2. However, without heat treatment, the value in S3 was higher than that in S1 and S2 but decreased as the drying temperature increased.

### 2.2. Radical Scavenging Activities and Reducing Power

**Figure 2:**
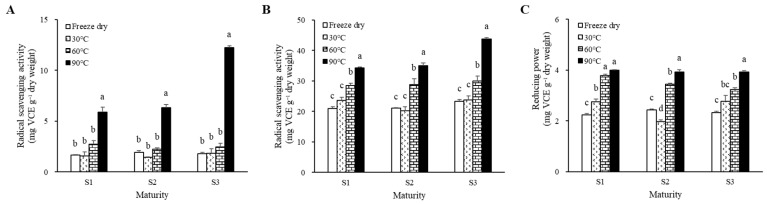
Radical scavenging activity of (**A**) DPPH and (**B**) ABTS and (**C**) reducing power evaluated using FRAP assay according to drying temperature of different maturities of *M. charantia* fruit. Different letters within each maturity stage indicate significant differences according to Tukey’s studentized test at *p* < 0.05.

In Figure 2, the changes in antioxidant activity in *M. charantia* dried at different temperatures were evaluated using 2,2-diphenyl-1-picrylhydrazyl (DPPH) and 2,2′-azino-bis (3-ethylbenzothiazoline-6-sulfonic acid (ABTS) radical scavenging activities and ferric-reducing antioxidant power (FRAP). In all growth stages of *M. charantia*, the radical scavenging activities and reducing power increased as the drying temperature increased and were significantly higher in the 90 °C heat-treated fruit than other treatments.

Similar variation patterns in the DPPH radical scavenging activity were observed between S1 and S2 after thermal treatments, whereas S3 showed distinct differences compared to the earlier stages. The DPPH radical scavenging activity of the 90 °C heat-treated sample in S3 was 6.8-fold higher than that of the freeze-dried (FD) sample, whereas it was 2.7- and 2.9-fold higher in S1 and S2, respectively. The changes of antioxidant activities of *M. charantia* at different stages showed a positive temperature-dependent increase in the ABTS radical scavenging activity and FRAP. Similar to DPPH radical scavenging activity, the 90 °C heat-treated S3 exhibited higher ABTS radical scavenging activity than the early stages. However, the FRAP did not show a clear difference between each maturity stage according to the temperature, in contrast to the radical scavenging assays.

### 2.3. Phenolic Contents

**Figure 3:**
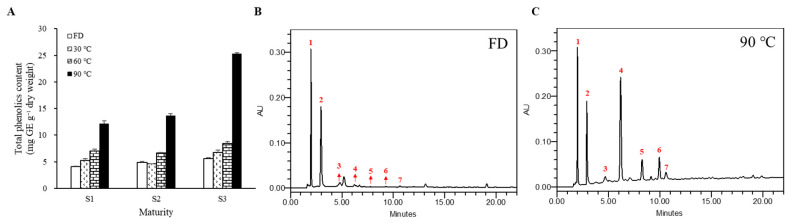
Changes of total phenolic content (**A**) in thermal processing of *M. charantia*. (**B**) Chromatograms of phenolic compounds in (**B**) freeze-dried (FD) and (**C**) 90 °C heat-treated S3 detected at 280 nm using HPLC analysis.

Figure 3A shows the effects of thermal processing on total phenolic content (TPC) in *M. charantia*. In nonthermal treatment, the TPC was 4.1, 4.9, and 5.6 mg gallic acid equivalent (GE)·g^−1^ dry weight (DW) in S1, S2, and S3, respectively. The TPC increased as the drying temperature increased, regardless of the stages after thermal treatment. In comparison to FD, the TPC increased with temperature in 60 °C and 90 °C heat-treated fruit, regardless of their stages. The higher total phenolic contents were exhibited at 90 °C heat-treated fruit in each stage, with 11.8 mg GE·g^−1^ DW in S1, 13.7 mg GE·g^−1^ DW in S2, and 25.3 mg GE·g^−1^ DW in S3.

In the HPLC chromatogram, seven distinct peaks, which are potential phenolic compounds, in high temperature-treated *M. charantia* were detected at 280 nm. The relative content of each compound among the thermal treatments was calculated on the bases of the total peak area in FD of S1 (Table 1). Furthermore, regardless of maturity, peaks 1 and 2 were major substances in FD. These contents increased significantly after high-temperature treatments. In the S3 samples, the contents of peaks 1 and 2 increased up to 30 °C and 60 °C and then decreased at higher temperatures. Interestingly, the content of peak 3 showed variations in patterns at the different growth stages. In samples of S1 and S3, the content slightly decreased at 30 °C and then increased with elevation in the drying temperature. The peak 3 contents of dried fruit at 90 °C were about twice as high as those of FD. Peaks 4–7 were not observed in FD *M. charantia* regardless of stages. However, the content of each compound continuously increased in *M. charantia* as the drying temperature increased, resulting in from 5 to 120 times higher content in the 90 °C heat-treated fruit compared to that in the FD at each stage.

### 2.4. Changes of Carotenoids and Chlorophylls

Table 2 shows the changes in the carotenoid and chlorophyll content of different maturities of *M. charantia* during thermal processing. Here, nine carotenoids and two chlorophylls were determined. In the nonthermal-treated fruit, the total content of the carotenoids increased as the fruit stage progressed. After thermal treatment, the total content of all stages of the fruits decreased continuously with the increasing drying temperatures. Three patterns were roughly observed after heat treatment for each carotenoid content variation. In all stages, the contents of carotenoid ester 1 and lutein decreased constantly with the increasing drying temperature in all stages, except carotenoid ester 1 did not exist in S3. The content of carotenoid ester 2 decreased as the growth stage progressed but was maintained with relatively small variation during thermal treatment. Regardless of the thermal treatment, carotenoids esters 3–7 and β-carotene were rarely or not detected in S1 and S2. Notably, these compounds were found in relatively large amounts in the FD of S3. However, at temperatures above 60 °C, the patterns demonstrated that each compound decomposed similarly. In the FD-treated fruit, the total chlorophyll content decreased with increasing fruit maturity stages. Moreover, no chlorophyll was found at the mature yellow stage in the fruit. After thermal treatment, the total chlorophyll content in S1 and S2 significantly decreased as the drying temperature increased. The content variation of chlorophyll a and b are responsible for the observed results.

### 2.5. Correlation between Antioxidant Activities and Physiochemicals

**Figure 4:**
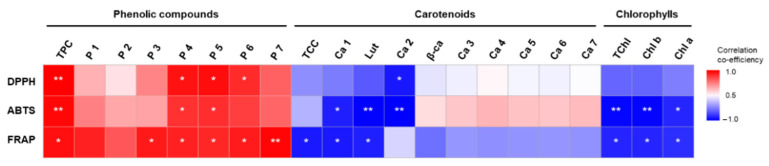
Correlation coefficients between antioxidant activities and phytochemicals in heat-processed *M. charantia*. TPC: total phenolic content; P1–P7: candidate phenolic compound 1–7; TCC: total carotenoid content; Ca1–Ca7: carotenoid ester 1–7; Lut: lutein; β-ca: β-carotene; TChl: total chlorophylls; Chl b: chlorophyll b; and Chl a: chlorophyll a. Asterisks indicate significance (* *p* < 0.05; ** *p* < 0.01) using Pearson’s correlation analysis.

Figure 4 shows the correlation between antioxidant activities and phenolic compounds, carotenoids, and chlorophylls. Overall, the antioxidant activities showed positive correlations with phenolics, whereas the antioxidant activities were negatively correlated with either carotenoids or chlorophylls. Furthermore, DPPH, ABTS, and FRAP activities showed a high correlation with TPC, exhibiting high correlation values (*r*) of 0.77, 0.72, and 0.70, respectively. These activities showed a significant correlation (*r* > 0.6, *p* < 0.05) with candidate phenolics 4–6, which significantly increased in the 90 °C heat-treated fruits.

## 3. Discussion

Color is an important indicator for judging fruit quality [27,28]. During ripening, the color of *M. charantia* is determined by the quality and quantity of natural pigments, such as the greenish contribution of chlorophylls and the yellowish contribution of carotenoids. According to current studies, the decrease in chlorophylls and the increases in carotenoids in *M. charantia* fruit during maturity strongly support the color variation patterns with low lightless and high redness with yellowness in mature yellow fruit than early stages. Color has also become an index to evaluate the quality of the processing, as pigment compounds can be decomposed or oxidized. The pigment changes during thermal processing are significantly affected by many factors, such as methods, time, and temperature [27,29]. In our results, the color of the hot-air-dried fruit was obviously different from that of the freeze-dried (Figure 1). The degradation of pigment compounds after thermal processing in *M. charantia,* regardless of the stages, should be responsible for these phenomena. On the other hand, it was found that browning occurred in all stages of the fruit treated at 90 °C, accompanied by mature yellow fruit treated at 30 °C. Similar results were reported in persimmon by Lim and Eom [10]. These are tentatively assumed to be the results of enzymatic and nonenzymatic browning. At a relatively low temperature (30 °C), the enzymatic browning occurs actively, causing discoloration. The moisture of the fruits dried fast and the enzyme was destructed at a relatively high temperature (90 °C). Nonenzymatic browning such as caramelization and the Maillard reaction probably happened due to the presence of amino acids and sugars in fruit at high temperatures [10]. Thus, it is important to determine the appropriated drying temperature to ensure the color quality of the fruit.

The significantly Increased antioxidant activities were shown in *M. charantia* after thermal processing, regardless of the fruit stages. The greater antioxidant activities were exhibited at higher drying temperatures (Figure 2). Interestingly, the changes in antioxidant activity of *M. charantia* at different growth stages showed different responses to heat treatment. Furthermore, for radical scavenging assays, more increased antioxidant activities were observed in high-temperature-treated fully matured fruit than in the earlier stages. The DPPH radical scavenging activity of the 90 °C heat-treated S3 was found to be 6.8-fold higher than that of the FD sample, while it was 2.7- and 2.9-fold higher in S1 and S2, respectively (Figure 2). However, the reducing power of the fruit did not show a clear difference between each maturity stage according to the temperature. Here, it is important to point out the characteristics of different antioxidant reactions among the colorimetric antioxidant assays. The DPPH assay tends to react with the hydrophobic compounds, the ABTS assay tends to screen both lipophilic and hydrophilic compounds, and the FRAP assay presents nonspecific properties [30,31]. The difference in antioxidant activity variation patterns among the three assays suggested that thermal processing induced the release of hydrophobic compounds and an increase in lipophilic compounds more than hydrophilic compounds.

The increase in antioxidant activities might be due to the variation of polyphenols in the matrix after thermal processing. The polyphenols are important antioxidant contributors [10,11,12,13] and the significantly increased total phenolic content in the thermally treated *M. charantia* can support the increased antioxidant activities, which were evaluated using three different assays (Figure 2 and Figure 3). A chromatogram at 280 nm is widely used to study the polyphenols because the absorption at this wavelength is suitable for detecting a large number of such compounds [32]. Based on the lambda max of the peaks at 280 nm in our data and the phenolic acid profiling of *M. charantia* from a previous report [24], the peaks we detected are considered to be phenolic compounds. The change of each phenolic compound from the heat treatment showed a significantly different pattern (Table 1). Our results demonstrated that peaks 4–6 were significantly increased by heat at all maturities, contributing to antioxidant activity. Although these peaks were increased by heat regardless of maturity, the increased levels were highest in S3, showing a strong correlation with the antioxidant activity. The quantitative increase may result from the thermal conversion of insoluble phenolic compounds into soluble form [33]. According to Horax et al. [24], several phenolic compounds were increased after the 60 °C oven-drying of *M. charantia* fruits, where the increased phenolics differed depending on the part of the fruit; mainly, the increased phenolics were gallic acid and catechin in the flesh, while they were gentisic acid and epicatechin in the inner tissue. It has been reported that food processing, such as heat treatment, can lead to polyphenol degradation from cellular structures, increasing phenolic compounds [33,34]. Thermal processing can also result in an enhanced extract ratio of the polyphenols. High-temperature treatment may have caused the decomposition of high-molecular phenolics into low-molecular phenolic compounds such as gallic acid and epicatechin [33,34]. Thus, our results connote that mature yellow fruits contain relatively more high-molecular phenolics or that low molecular phenolics are easily degraded in mature fruit.

Carotenoids are also known to exhibit antioxidant activity [35,36]. Previous studies found that carotenoid variation accumulates during *M. charantia* maturation [37,38] and that several carotenoids only existed in the mature yellow stage [39]. These results are consistent with our finding that five carotenoids are generated only in S3 of *M. charantia* (Table 2). Regardless of the fruit growth stage, the content of carotenoids decreases with higher processing temperatures. The destruction of these carotenoids is similar to the results of previous studies showing that carotenoids are decomposed by the oxidation of oxygen at high temperatures [23]. Interestingly, although carotenoids are known to be heat-sensitive compounds, carotenoid ester 2, which maintained a relatively high content despite heat treatment, was observed during heat treatment. This may be due to the different thermal stabilities according to the structure of the carotenoid [40,41].

The correlation coefficiency analysis suggested a high positive correlation between the antioxidant activities and phenolics but no or negative correlation between the antioxidant activities and carotenoids or chlorophylls. For individual candidate phenolic compounsd detected at 280 nm, peaks 4 and 5 showed a significant positive correlation with three antioxidant activity assays. Similarly, phenolic compounds in *M. charantia* have previously been studied as potential antioxidants [42,43]. Although thermal treatment has a negative effect on carotenoid preservation during *M. charantia* fruit processing, our results suggest that the maturity of the fruit and heating temperature are critical factors enhancing phenolic compounds and antioxidant activity.

## 4. Materials and Methods

### 4.1. Plant Materials

The *M. charantia* plants were cultivated in the greenhouse of Kyung Hee university (Yongin si, Republic of Korea). These plants were planted in horticultural soil (Baroker, Seoulbio Co., Eumseong, Republic of Korea) mixed with perlite (GFC. Co., Ltd. Hongseong, Republic of Korea) on 10 May 2021. The fruits of the *M. charantia* were harvested at three different maturity stages: S1, immature green with undeveloped seeds (15.52 ± 0.61 cm of length); S2, mature green with fully developed seeds (23.14 ± 1.10 cm of length); S3, mature yellow with red ripe seeds (26.54 ± 0.64 cm of length). We randomly harvested 20 individuals in the growth stage from 15 plants three times in July and August 2021. The experiment was carried out with a total of four repetitions with 5 objects as one repetition.

The fruits were washed with distilled water and dried using a paper towel. The fruit samples were cross sectioned at a thickness of 1 cm. The skin, flesh, and inner tissues were obtained and the seeds were removed (Figure 1A). The separated fruits were immediately dried after each harvest without storage. Batches of the samples were freeze-dried in a freeze dryer as a control group. For thermal processing, the samples were hot-air dried using a dry machine (Koencon Co., Ltd., Hanam, Republic of Korea) until the water content was <10% at 30, 60, and 90 °C for 3 days, respectively. The dried samples were pulverized using a commercial mixer and sieved with a 100-mesh size.

### 4.2. Color Measurement

The colors of the ground *M. charantia* samples were obtained by using a color analyzer (Lutron Electronics, Inc., Coopersburg, PA, USA). The acquired RGB values were converted into L* (lightness), a* (greenness to redness), and b* (blueness to yellowness) using OpenRGB v. 2.30.10125 software.

### 4.3. Sample Extraction

#### 4.3.1. Preparation of Extract for the Determination of Antioxidant Activities, Total Phenolic Content, and Content of Individual Phenolic Compound

The extraction method was performed as previously described methods [24,44] with some modifications. The dried samples (50 mg) were immersed in 1 mL of 80% ethanol and placed in a shaking incubator at 24 °C for 8 h after 1 h sonication. Later, the mixture was centrifuged at 12,000× *g* for 10 min and the supernatant was collected for further analysis.

#### 4.3.2. Preparation of Extract for Carotenoid and Chlorophyll Analysis

The extraction method was modified slightly from that described by Lim and Eom [10]. The ground sample was extracted with 350 μL of methanol and, later, 700 μL of chloroform was added to it. After vortexing, 350 μL of 10% sodium chloride (NaCl) was added to the mixture, which was then centrifuged at 8000× *g* for 5 min. The chloroform phase was separated from the mixture in fresh tubes. Potassium hydroxide (350 μL, 1 N) was added to the chloroform phase and the mixture was heated in the dark for 30 min. The mixture was centrifuged after adding 10% NaCl and the chloroform phase was collected. The collected phase was washed with additional 10% NaCl (700 μL) to remove the KOH. The chloroform phase (500 μL) was centrifuged and 800 μL of ethyl acetate was added to the collected phase. The final mixture was filtered using a 0.45 μm syringe filter (Futecs Co., Ltd., Daejeon, Republic of Korea) and the filtrate was used for carotenoid and chlorophyll content analysis.

### 4.4. Colormetric Assays of Antioxidant Activities

The antioxidant assays of the *M. charantia* extract following thermal processing were measured using the 2,2-diphenyl-1-picrylhydrazyl (DPPH) and 2,2′-azino-bis (3-ethylbenzothiazoline-6-sulfonic acid (ABTS) radical scavenging activity assays. The antioxidant assays were performed as described by the method of Lim and Eom [10] with some modifications. For DPPH radical scavenging activity, the sample or standard (17 μL) was mixed with 983 μL of DPPH solution. The absorbance of the mixture was measured at 517 nm after the reaction in the dark for 30 min. The DPPH solution was adjusted by 0.65 ± 0.02 in the absorbance value with 80% methanol at 517 nm. For ABTS radical scavenging activity, 10 mM ABTS dissolved in DMSO was mixed in a 1:4 ratio with 8 mM of 2,2′-Azobis (2-amidinopropane) dihydrochloride dissolved in 1X phosphate-buffered saline (PBS). The mixture was heated at 70 °C for 40 min. The ABTS solution was filtered using a 0.45 μm syringe filter and adjusted to a 0.65 ± 0.02 in absorbance value with 1X PBS at 734 nm. The solution was added to the sample (20 μL) and measured at 734 nm after a 10 min incubation at room temperature. The DPPH and ABTS radical scavenging activities were expressed as milligrams of vitamin C equivalents (VCE) per gram of DW.

The ferric-reducing antioxidant power assay was performed as described by the method of Lim and Eom [10] with minor modifications. A solution of 300 mM acetate buffer (pH 3.6) was prepared by dissolving 3.1 g sodium acetate trihydrate and 16 mL acetic acid in 1 L of distilled water. The 10 mM 2,4,6-tripyridyl-s-triazine (TPTZ) in 40 mM hydrochloric acid were and 20 mM ferric chloride (FeCl_3_·6H_2_O) solution prepared. The FRAP solution was prepared by mixing acetate buffer, TPTZ solution, and FeCl_3_·6H_2_O in a 10:1:1 ratio. The 950 μL of FRAP solution was added to 50 μL of sample extract and reacted in the dark for 30 min. The absorbance of the mixture was measured at 593 nm. The FRAP was expressed as milligrams VCE per gram DW.

### 4.5. Measurement of Total Phenolic Content

The total phenolic content was determined in accordance with the Folin–Ciocalteu method of Lim et al. [45] with some modifications. The sample extract (50 μL) was added to 650 μL of distilled water. The 50 μL of Folin–Ciocalteu phenol reagent was immediately added and mixed. After 6 min of incubation, 500 μL of 7% sodium carbonate (Na_2_CO_3_) was added and reacted at room temperature for 90 min.

### 4.6. Determination of Individual Phenolic Compound

The 0.2 mL of the sample extract was diluted with 0.8 mL of 80% ethanol. The diluted extract was filtered through a 0.45 μm syringe filter. The filtrate was analyzed using reverse-phase HPLC (Waters 2695 Alliance HPLC; Bischoff, Leonberg, Germany) with a prontosil column (120-5-C18-SH, 5 μm, 150 × 4.6 mm; Bischoff, Leonberg, Germany) as previously described methods [18,24]. The mobile phase consisted of (A) water with 0.1% formic acid and (B) acetonitrile. The gradient elution was as follows: 0–23 min, 1–20% B; 23–45 min, 20–60% B; 45–46 min, 60% B; 46–47 min, 60–1% B; and 47–49 min, 1% B. The flow rate of the mobile phase was 1.0 mL·min^−1^ and the injection volume of the sample was 10 μL. The peaks were detected at 280 nm using the Waters 996 photodiode array detector (Waters Inc., Milford, MA, USA).

### 4.7. Determination of Carotenoids

The saponified sample extract was used for the HPLC analysis. The analysis was performed using Waters 2695 Alliance HPLC as in the previously described method [10]. The column used prontosil 120-5-C18-SH 5.0 μm (4.6 × 250 mm, Bischoff, Leonberg, Germany). Mobile phase A consisted of 90% acetonitrile with 0.1% formic acid and mobile phase B consisted of ethyl acetate with 0.1% formic acid. The gradient was as follows: 0–10 min, 0–60% B; 10–25 min, 60% B; 25–26 min, 60–0% B; and 26–27 min, 100% A. The flow rate was 1.0 mL·min^−1^. The injection volume was 10 μL. The peaks were detected at 445 nm using a Waters 996 photodiode array detector (Waters Inc., Milford, MA, USA). The quantitative data of the carotenoid was expressed as relative content based on the total carotenoid of the freeze-dried S1 sample. The chlorophyll data were expressed as relative content of the total chlorophyll of freeze-dried S1 sample.

### 4.8. Statistical Analysis

All the samples were performed in three replicates and expressed as the mean and standard error. The data were analyzed using SAS software (Enterprise Guide 7.1 version; SAS Institute Inc., Cary, NC, USA). A one-way analysis of variance was performed to assess the differences between the mean values using a Fisher’s least significant difference (LSD) test. The significant differences among the experimental treatments were evaluated using Tukey’s studentized test (HSD) at *p* < 0.05. The relationship between the antioxidant activity and component contents under each treatment was analyzed using Pearson’s correlation coefficients. 

## 5. Conclusions

This study investigated the effect of thermal processing on the color, antioxidant activities, phenolics, carotenoids, and chlorophylls in the growth stage of *M. charantia* fruit. Thermal treatment led to distinct color changes in different maturities of *M. charantia*. The changes from green to yellow in greenish fruits were caused by the degradation of chlorophylls, whereas the changes from yellow to brown in mature fruit were caused by two different reactions, indicating enzymatic browning by low-temperature dry processing and nonenzymatic browning by high-temperature dry processing. After thermal treatment, antioxidant activities increased in fruit with increasing drying temperatures, regardless of the maturity stage of the fruit. The maturity of *M. charantia* affected the changes in radical scavenging activity after heat treatment, whereas it had no effect on reducing power. Due to the different mechanisms of each assay, these results are tentatively explained by the release of hydrophobic compounds and the increase in lipophilic compounds. The total phenolic contents were also significantly increased in each maturity of *M. charantia* after thermal treatment. Particularly, the highest content was observed in the 90 °C heat-treated S3 fruit, which is extremely higher than the 90 °C heat-treated S1 and S2. These results may be due to the significantly increased content of the candidate phenolic compounds 4, 5, and 6, which were detected at 280 nm using HPLC. However, after thermal processing, both carotenoids and chlorophylls were significantly decreased. The correlation coefficiency test between antioxidant activity and bioactive compounds suggested that the antioxidant activities of *M. charantia* were mainly contributed to by phenolic compounds. Although thermal processing induced the decrease in the carotenoid of *M. charantia* fruit, our results suggest that the maturity of fruit and the processing temperature are the critical factors enhancing phenolic compounds and antioxidant activity and that mature yellow fruit is better for consumption after using thermal processing. Overall, these results suggest that thermal processing at a high temperature can be usefully applied in industries of health supplements and nutraceuticals of *M. charantia* fruit and provide an optimized harvesting time and processing method for the development of functional foods.

## Figures and Tables

**Table 1:** The change in the content (%) of seven candidate phenolic compounds according to the drying temperature of fruits.

Peak	RT (min)	λmax	S1				S2				S3				LSD
			FD	30 °C	60 °C	90 °C	FD	30 °C	60 °C	90 °C	FD	30 °C	60 °C	90 °C	
1	1.99	217.7/273.1	38.86 f	48.09 de	43.54 ef	72.87 a	44.84 def	23.64 g	61.01 bc	64.13 ab	47.99 de	67.98 a	59.07 bc	51.06 cd	1.88
2	2.9	274.3	50.13 cd	49.15 cd	41.80 d	80.37 a	46.15 cd	21.18 e	70.28 b	68.03 b	54.93 c	73.14 ab	69.52 b	44.25 d	2.35
3	4.69	260.1	8.21 d	7.90 de	11.20 c	16.12 a	7.81 de	1.80 g	0.00 g	13.45 b	5.81 ef	3.82 f	10.35 c	16.83 a	0.44
4	6.18	296.1	1.88 de	0.45 e	4.69 de	46.24 b	1.03 e	0.60 e	2.42 de	29.58 c	2.12 de	1.53 de	6.57 d	92.01 a	1.25
5	8.26	263.7	0.12 e	0.32 de	1.14 d	9.10 b	0.27 de	0.14 e	0.36 de	6.57 c	0.13 e	0.20 de	0.75 de	21.26 a	0.22
6	9.94	289.7	0.25 e	0.35 e	0.33 e	9.97 b	0.28 e	0.37 e	0.21 e	8.40 c	0.15 e	0.74 d	0.58 d	17.78 a	0.06
7	10.58	213.0/257.7	0.54 f	1.19 e	2.15 d	6.68 b	1.50 de	1.72 de	1.11 e	5.95 c	1.46 de	1.20 e	1.46 de	8.00 a	0.16
Total			100.00	107.45	104.82	241.35	101.88	49.45	135.38	196.09	112.60	148.60	148.30	251.19	

The relative content of each compound among the thermal treatments was calculated in the bases of total peak area in FD of S1. Alphabetical letters within a row indicate significant difference in Tukey’s studentized test at *p* < 0.05. Peak number corresponds to the peak number of Figure 3B chromatograms.

**Table 2:** Carotenoid and chlorophyll content (%) by drying temperature of *M. charantia* at different maturity stages.

Peak	Compounds	Rt (min)	λmax	S1				S2				S3				LSD
				FD	30 °C	60 °C	90 °C	FD	30 °C	60 °C	90 °C	FD	30 °C	60 °C	90 °C	
Carotenoid																
1	Carotenoid ester 1	8.59	412.8/439.4	3.63 a	2.81 c	n.d.	n.d.	3.06 b	1.80 d	n.d.	n.d.	n.d.	n.d.	n.d.	n.d.	0.22
2	Lutein	12.4	462.4/487.9	47.15 a	30.65 b	11.35 d	4.35 e	32.34 b	27.61 c	3.24 e	4.10 e	3.35 e	n.d.	n.d.	n.d.	2.99
3	Carotenoid ester 2	13.5	418.8	45.88 a	42.33 abc	39.81 bcd	39.89 bcd	37.37 cde	38.87 bcd	41.34 ab	39.38 bcd	35.71 de	35.13 de	35.70 de	33.73 e	5.43
4	β-carotene	19.7	427.3/451.5	3.34 b	n.d.	n.d.	n.d.	n.d.	n.d.	n.d.	n.d.	11.25 a	2.07 c	n.d.	n.d.	0.78
5	Carotenoid ester 3	26.1	445.1/481.8	n.d.	n.d.	n.d.	n.d.	n.d.	n.d.	n.d.	n.d.	34.56 a	3.53 b	n.d.	n.d.	1.09
6	Carotenoid ester 4	29.03	447.8/487.9	n.d.	n.d.	n.d.	n.d.	n.d.	n.d.	n.d.	n.d.	13.88 a	4.66 b	n.d.	n.d.	0.85
7	Carotenoid ester 5	30.2	446.6/473.3	n.d.	n.d.	n.d.	n.d.	n.d.	n.d.	n.d.	n.d.	10.12 a	1.66 b	n.d.	n.d.	0.59
8	Carotenoid ester 6	31.0	453.9/478.1	n.d.	n.d.	n.d.	n.d.	n.d.	n.d.	n.d.	n.d.	36.30 a	4.59 b	n.d.	n.d.	1.95
9	Carotenoid ester 7	34.2	452.7/483	n.d.	n.d.	n.d.	n.d.	n.d.	n.d.	n.d.	n.d.	27.22 a	6.62 b	n.d.	n.d.	1.10
	Total carotenoid			100.00	75.79	51.16	44.24	72.77	68.29	44.58	43.48	172.38	58.26	37.91	33.73	
Chlorophyll																
10	Chlorophyll b	14.1	457.5/643.2	88.17 a	68.55 b	23.52 d	2.56 f	66.31 b	51.81 c	6.28 e	n.d.	n.d.	n.d.	n.d.	n.d.	2.92
11	Chlorophyll a	15	429.6/660.4	11.83 a	5.77 b	1.63 d	n.d.	5.39 b	4.34 c	n.d.	n.d.	n.d.	n.d.	n.d.	n.d.	0.95
	Total chlorophyll			100.00	74.32	25.15	2.56	71.69	56.15	6.28	n.d.	n.d.	n.d.	n.d.	n.d.	

The relative content of each compound among the thermal treatments was calculated in the bases of total peak areas in FD of S1. Alphabetical letters within a row indicate significant difference in Tukey’s studentized test at *p* < 0.05. n.d. indicates not detected.

## Data Availability

All of the data is contained within the article.

## Sample Availability

Samples are not available from the authors.
